# Psychometric properties of the 10-item Conner-Davidson resilience scale on toxic chemical-exposed workers in South Korea

**DOI:** 10.1186/s40557-018-0265-5

**Published:** 2018-08-13

**Authors:** Gab-Sik Shin, Kyeong-Sook Choi, Kyoung Sook Jeong, Young-Sun Min, Yeon-Soon Ahn, Min-Gi Kim

**Affiliations:** 1 Departments of Occupational and Environmental Medicine, Dongguk University GyeongjuHospital, 87 Dongdae-ro, Seokjang-dong, Gyeongju-si, Gyeongbuk 780-350 Republic of Korea; 2 Department of Neuropsychiatry, Eulji University School of Medicine, Dunsanseo-ro, Seo-Gu, Daejeon, Republic of Korea; 3 Department of Occupational and Environmental Medicine, Hallym University Sacred Heart Hospital, 22 Gwanpyeong-ro 170 beon-gil, Anyangsi, Gyeonggido 14068 South Korea; 4 Department of Preventive Medicine, Yonsei University Wonju College of Medicine, 20, Ilsan-ro, Wonju, Gangwon 220-701 South Korea; 5 Department of Occupational Medicine, Dankook University Cheonan Hospital, Cheonan-si, Chungnam Republic of Korea

**Keywords:** Hydrogen-fluoride, Post-traumatic stress disorder, 10-item Connor-Davidson resilience scale

## Abstract

**Background:**

Resilient individuals have a comprehensive ability to adapt to various life circumstances. Psychological resilience predicts an individual’s physiological response to stress. The 10-item Connor-Davidson Resilience Scale (CD-RISC) is a widely used measure to quantify the level of self-perceived resilience. This study examined the psychometric properties of a Korean version of the 10-item Connor-Davidson Resilience Scale (10-item K-CD-RISC) on workers in Gumi, South Korea, exposed to hydrofluoric acid (HF).

**Methods:**

The questionnaires included the 10-item K-CD-RISC and Beck Anxiety Inventor (BAI), the Impact of Event Scale-Revised-Korean version (IES-R-K), the Rosen-berg Self Esteem Scale (RSES), the Center for Epidemiologic Studies-Depression Scale (CES-D), and the Perceived Stress Scale (PSS). These were randomly distributed at 237 workplaces near the HF-spill site, in the Gumi 4 complex. The responses of 991 (67.3%) workers were analyzed.

**Results:**

The exploratory factor analysis shown that a single-factor model was consistent with the original design of the 10-item CD-RISC. The scale also demonstrated good internal consistency (Cronbach’s alpha = 0.95). Scores on the scale reflected different levels of resilience with respect to personal factors (age, gender, marital status, and education and income levels) that are thought to be differentiated. Differences of resilience were also reflected by psychiatric symptoms (anxiety and depression). Moreover, the total score of scale positively correlated with RSES, whereas the IES-R-K, BAI, CES-D, and the PSS negatively correlated with the 10-item K-CD-RISC.

**Conclusions:**

The 10-item K-CD-RISC has good psychometric properties and is applicable for victims exposed to noxious chemical such as HF.

## Background

Resilience is defined as a protective factor against mental problems and a dynamic process for adapting to changes in the various facets of life [1, 2]. As resilience can be used to indicate the successful adaptation of an individual to trauma, this concept has garnered increasing interest in the fields of trauma-related research and practice in recent years [4–6]. Resilience is reported to play an influential role in buffering the adverse effects of traumatic events and protecting against the development of posttraumatic stress disorder (PTSD) or against major depressive disorder (MDD) [4, 7–9].

Resilience can be viewed as a measurement of stress-coping ability [3]. Various tools are available for measuring resilience [10, 11]. The Connor-Davidson Resilience Scale (CD-RISC), a metric widely used to quantify the level of self-perceived resilience [3], has the ability to identify target groups for intervention against PTSD development [12]. Furthermore, CD-RISC is one of the most widely validated scales that crucially explains the concept of resilience [13], and it has been translated into many languages across a wide range of groups and populations [14, 15]. The scale has been validated across various samples of young adults [16], teenagers [17], young women [18], nurses [19], graduate students [20] as well as the general populations [21]. In addition, the CD-RISC has been employed in studying of natural disasters in Sichuan [15], Japan [22] and Turkey [23], and in New Zealand [24] earthquakes and other environmental accidents such as the Deep-Water Horizon oil spill [25]. As such, CD-RISC has been widely used in research to objectively quantify resilience, and the reliability and validity of this scale has been verified in numerous studies. These studies provide the preliminary evidence to confirm the acceptability and applicability of the CD-RISC on objects of resilience studies.

Originally structured in five dimensions, the factor structure of the CD-RISC has revealed certain limitations related to multi-dimensional concept proposals [26]. Thus, a new 10-item version of CD-RISC was developed, which resulted in a stable scale with excellent psychometric properties [13].

Not many studies have evaluated the psychometric properties of victims exposed to toxic chemicals using the 10-item CD-RISC. Particularly, there has been no study on HF-exposed victims. This study was therefore designed to examine the psychometric properties of the 10-item K-CD-RISC in workers exposed to HF in Gumi, South Korea.

## Methods

### Participants

Of the 237 workplaces in the Gumi 4 complex, Korea, half were randomly selected and grouped based on the distances from the accident site (< 0.5 Km, 0.5–1.0 Km, ≥ 1.0 Km). Ten months after the HF-spill accident, between 15 July 2013 and 26 July 2013, 10 researchers visited the selected sites to conduct surveys. Surveys were conducted only for workers at the randomly selected 118 workplaces and who were willing to participate in the study. In total, 1480 workers at 93 workplaces responded to the survey questionnaire. Among the respondents, responses of 991 (67.3%) workers who accurately responded to the questionnaire were analyzed.

### Measures

#### 10-item K-CD-RISC

The 10-item CD-RISC was extracted from the original 25-item CD-RISC [13]. Reliability and validity of the 10-item K-CD-RISC in the Korean population is well-documented [27]. Each item is rated on a 5-point Likert scale from 0 (‘not true at all’) to 4 (‘true nearly all the time’). The description of each item is presented in Table 1. Total scores were obtained by summing all responses and ranged from 0 to 40, with higher scores reflecting greater resilience [15].

**Table 1:** Mean 10-item K-CD-RISC scores and standard deviations (SD) for sociodemographic categories

Variable		N (%)	Mean ± SD	*p*-value
Gender	Men	768 (79.8)	26.25 ± 7.65	< 0.01
	Women	194 (20.2)	22.61 ± 7.15	
Marriage	Single	530 (53.5)	24.59 ± 7.39	0.01
	Married	461 (46.5)	26.25 ± 7.97	
Age (years)	< 20	17 (1.7)	22.77 ± 8.59	0.01
	20–29	299 (30.5)	24.74 ± 7.82	
	30–39	420 (42.9)	25.23 ± 8.04	
	40–49	190 (19.4)	27.10 ± 6.85	
	50–59	49 (5.0)	26.69 ± 6.47	
Education	Less than high school	413 (42.2)	24.91 ± 8.05	0.04
	College	308 (31.5)	24.44 ± 7.28	
	University	258 (26.4)	26.47 ± 7.47	
Salary (US dollar/month)	< 2000	345 (38.4)	23.78 ± 8.14	< 0.01
	2000–3000	371 (41.3)	26.06 ± 7.13	
	≥ 3000	183 (20.4)	26.70 ± 7.24	

**p for trend < 0.05

#### Impact of event scale-revised (IES-R)

The IES-R is composed of 22-items that measure symptoms of intrusion, avoidance and numbing, and hyperarousal with respect to a traumatic event. All items of IES-R were in Korean. Each item consists of a 5-point Likert scale from 0 (‘not at all’) to 4 (‘very much’). The maximum score is 88, which indicates the worst PTSD symptom state. A cut-off value of 24 identified the participants with more than moderate PTSD symptoms [28].

#### The Beck anxiety inventory (BAI)

The BAI is a 21-items self-report questionnaire that lists symptoms of anxiety. The respondent rates each symptom to the extent it has bothered him/her in the past week. The symptoms are rated on a 4-point Likert scale, ranging from 0 (‘not at all’) to 3 (‘severely’). A cut-off value of 21 for the BAI identified the participants with more than moderate anxiety symptoms [29, 30].

#### The Center for Epidemiologic Studies of depression scale (CES-D)

The CES-D is a self-report measure consisting of 20-items, with response options for each item reflecting varying levels of depression symptoms. It ranges from ‘rarely or none of the time’ (0 point) to ‘most or all of the times’ (3 points). The total score ranges from 0 to 60, in which a higher score indicates more severe depressive symptoms. A cut-off value of 20 for the CES-D identified the participants with more than moderate depressive symptoms [31, 32].

#### Rosenberg self-esteem scale (RSES)

This is a 10-item scale that assesses global self-worth by measuring both positive and negative feelings about the self. The scale is believed to be uni-dimensional. All items are answered using a 4-point Likert scale ranging from strongly agree (4 points) to strongly disagree (1 point). Total scores range from 10 to 40, with higher scores representing higher self-esteem [33].

#### Perceived stress scale (PSS)

The PSS is a self-report assessment with the current version having 10 items. The PSS uses the 5-point Likert scale to measure the degree to which individuals perceived their daily life as being stressful during the past month (0 = never and 4 = very often). Total scores range from 0 to 40. Higher scores on the PSS represent higher levels of perceived stress [34].

### Data analysis

Means and standard deviation (SD) were used for simple descriptive statistics. The factor structure of the 10-item K-CD-RISC was investigated using the exploratory factor analysis and the maximum likelihood method. Parallel analysis was employed to determine the number of factors to be retained. As recommended in the report by O’Connor [35], 100 random datasets were generated using a procedure in SPSS, and the 95th percentiles of the eigenvalues were obtained from actual datasets. Factors were retained if the eigenvalue from the actual data was greater than the corresponding eigenvalue from random data [15]. We also measured the reliability related to internal consistency by the Cronbach’s alpha coefficient (Cronbach’s α).

Construct validity was performed by comparing the 10-item K-CD-RISC scores across various subgroups on the basis of sociodemographic characteristics and psychiatric symptom group (depression, anxiety, probable PTSD) using analyses of one-way ANOVA with post hoc testing using Duncan’s multiple range test and Student’s t-test, depending on the number of categories. Convergent validity among the 10-item K-CD-RISC and other assessments (BAI, IES-R, PSS, RSES, and CES-D) was examined by determining Pearson’s correlation coefficients.

Statistical analyses were performed using the SPSS 20.0 for Windows software package (SPSS, Chicago, IL, USA). All reported *p* values were two-tailed, and values less than 0.05 were considered to be statistically significant.

## Results

### Characteristics of study population

Majority of the subjects included in this study were male (79.8%), and two-thirds of the subjects were over 30 years of age (67.8%). Approximately half were married, more than half the subjects were educated above college level, and 61.6% of the respondents received more than US $ 2000 per month. Most subjects had no mental disease.

Detailed characteristics of the study population are described in Table 1 and Table 2.

**Table 2:** Mean 10-item K-CD-RISC scores and standard deviations (SD) for trauma-related factors by HF spill

Variable		N (%)	Mean ± SD	*p*-value
Psychiatric diseases	No	980 (98.9)	25.54 ± 9.43	0.01
	Yes	11 (1.1)	19.27 ± 7.50	
Current symptoms associated with HF exposure	No	920 (92.9)	25.54 ± 7.65	0.30
	Yes	70 (7.1)	24.54 ± 8.45	
Perceived exposure rate of HF	No	171 (20.8)	25.12 ± 8.51	0.85
	Mild	457 (55.5)	25.71 ± 7.19	
	Middle	127 (15.4)	25.47 ± 7.23	
	High	69 (8.4)	25.70 ± 7.39	

### Factor structure

By using the principal components analysis, the first two eigenvalues from the actual dataset of the resilience scores were determined to be 6.9 and 0.69 Based on factor analysis results, we extracted a single-factor model. The resilience factor accounted for 69.43% of the total variance of the study population. The factor-loading matrix for the resilience score is presented in Table 3. All items exhibited silent factor loading (higher than 0.40) on the latent variable. These findings suggest that the 10-item K-CD-RISC has good structure validity (Table 3).

**Table 3:** Factor analysis of the 10-item K-CD-RISC

Item	Description	Factor loading
Item 1	Able to adapt to adapt to change	0.779
Item 2	Can deal with whatever comes	0.829
Item 3	Tries to see humorous side of problems	0.785
Item 4	Coping with stress can strengthen me	0.809
Item 5	Tend to bounce back after illness of hardship	0.847
Item 6	Can achieve goals despite obstacles	0.869
Item 7	Can stay focused under pressure	0.823
Item 8	Not easily discouraged by failure	0.850
Item 9	Thinks of self as strong person	0.886
Item 10	Can handle unpleasant feeling	0.825
Eigenvalue		6.90
Percentage of variance explained		69.43

### Reliability

Based on the single-factor model, the reliability related to internal consistency (measured by Cronbach’s α) was 0.95 for the whole resilience score.

### Construct validity

#### Subgroup comparisons

The resilience score (*M =* 26.25, *SD =* 7.65) of men was higher than that of women (*M =* 22.61, *SD =* 7.15), and the resilience score of married participants (*M =* 26.25, *SD =* 7.97) was higher than that of single participants (*M =* 24.59, *SD =* 7.39).

The results of one-way ANOVA indicate that mean resilience scores were significantly different for age, education, and salary groups. The result of Duncan’s multiple range post hoc test indicates a mean resilience score of 20–29 year-old participants to be lower than that of 40–49 year-old participants (*p* <  0.01). In addition, the mean of resilience score of university-graduated participants was higher than that of high school under-graduate participants (*p* <  0.01). Moreover, the mean resilience score of participants with salary < 2000 US dollar (USD) was lower than that of participants with salary 2000–3000 USD and ≥ 3000 USD (Table 1).

#### Relationship between 10-item K-CD-RISC scores and trauma-related factors by HF spill

Participants with psychiatric disease (*M =* 19.27, *SD =* 7.50) had a lower resilience score than participants without psychiatric disease (*M =* 25.54, *SD =* 9.43). The mean resilience scores were not significantly different between participants afflicted with or without HF associated symptoms and did not differ with perceived exposure rate (Table 2).

#### Relationship between 10-item K-CD-RISC scores and psychiatric symptom

The mean resilience score of participants with BAI ≥ 21.0 (*M* = 19.94, *SD* = 7.31) was significantly lower than that of BAI <  20.9 workers (*M* = 25.67, *SD* = 7.64). The mean resilience score of participants with CES-D ≥ 20 (*M* = 21.73, *SD* = 7.42) was significantly lower than that of CES-D <  19.9 (*M* = 26.19, *SD* = 7.31). The mean resilience score was not significantly different according to level of PTSD symptom (Table 4).

**Table 4:** Mean 10-item K-CD-RISC scores and standard deviations (SD) by psychiatric symptom group

Variable		N (%)	Mean ± SD	*p*-value
IES-R	< 23.9	844 (87.0)	25.66 ± 7.79	0.09
	≥ 24.0	126 (13.0)	24.42 ± 7.31	
BAI	< 20.9	911 (95.0)	25.67 ± 7.64	< 0.01
	≥ 21.0	48 (5.0)	19.94 ± 7.58	
CES-D	< 19.9	810 (90.1)	26.19 ± 7.31	< 0.01
	≥ 20.0	89 (9.9)	21.73 ± 7.42	

*IES-R* Impact of Event Scale-Revised, *BAI* Beck Anxiety Inventory, *CES-D* The Center for Epidemiologic Studies of Depression Scale

#### Relationship between 10-item K-CD-RISC scores and other psychological measures

Convergent validity was assessed by comparing the 10-item CD-RISC resilience score with the RSES, BAI, CES-D, IES-R, and PSS results. The total resilience score positively correlated with RSES (*r* = 0.51, *p* <  0.01) and negatively correlated with IES-R (*r* = − 0.11, *p* <  0.01), BAI (*r* = − 0.23, *p* <  0.01), CES-D (*r* = − 0.17, *p* <  0.01), and PSS (*r* = − 0.36, *p* < 0.01) outcomes (Table 5).

**Table 5:** Correlations between the 10-item K-CD-RISC and related assessments

	K-CD-RISC 10	RSES	IES-R	BAI	CES-D	PSS
K-CD-RISC 10	–					
RSES	0.511^a^	–				
IES-R	− 0.111^a^	− 0.324^a^	–			
BAI	− 0.228^a^	− 0.392^a^	0.556^a^	–		
CES-D	− 0.169^a^	− 0.330^a^	0.450^a^	0.653^a^	–	
PSS	− 0.358^a^	− 0.547^a^	0.382^a^	0.444^a^	0.376^a^	–

^a^correlation is significant at the 0.01 level (2-tailed). K-CD-RISC 10: Korean Version of the 10-item Conner-Davidson Resilience Scale, *RSES* Rosenberg Self-Esteem Scale, *IES-R* Impact of Event Scale-revised, *BAI* Beck Anxiety Inventory, *CES-D* The Center for Epidemiologic Studies of Depression Scale, *PSS* Perceived Stress Scale

## Discussion

The 10-item CD-RISC has been verified in terms of reliability and validity by various groups and countries. This study examined the psychometric properties of the 10-item K-CD-RISC in workers exposed to noxious chemicals.

Exploratory factor analysis where all items exhibited notable factor loading (higher than 0.40) on the latent variable reveals that the data could be well represented by a single-factor model, which is consistent with the original design of the 10-item CD-RISC and explains that the 10-item K-CD-RISC is a unidimensional measurement of resilience. Furthermore, the scale demonstrated good internal consistency in this study (Cronbach’s α value of 0.95), which is higher than 0.91 of the China Sichuan earthquake study, indicating that the measurement error of the 10-item K-CD-RISC is small, and the 10-item K-CD-RISC has sufficiently high reliability to provide confidence in interpreting the score. Based on these analysis, we confirm that the 10-item K-CD-RISC can be employed to assess resilience in Korea workers.

Furthermore, this study found that the psychiatric symptom group rated higher scores on the IES-R, BAI, and CES-D, and lower scores on the 10-item K-CD-RISC. This finding suggests that scores on the 10-item K-CD-RISC reflect different levels of resilience in populations that are thought to be differentiated by psychiatric disease.

According to the correlation between the 10-item K-CD-RISC and related assessments in this study, the scale scores were positively correlated with RSES and negatively correlated with IES-R, BAI, CES-D, and PSS results. Although the magnitude of the R-value is not statistically large, the correlations between the 10-item K-CD-RISC and related assessments are well explained in the directional aspect.

These major findings provide authentic evidence that the 10-item K-CD-RISC is a reliable and valid measurement of resilience. Furthermore, there is very limited literature on psychometric properties, and particularly there is no literature on victims exposed to HF.

This study has several limitations. First, this study was conducted 10 months after the HF-spill accident. The IES-R well reflects the impact of the near-time event. The results inadequately reveal that resilience is associated with PTSD symptoms. Specifically, only the tendency of resilience based on the severity of PTSD symptoms was confirmed; the difference of resilience score was not large with no statistical significance. Consideration of timing of the study seems essential for future researches [36–39] and would be required to confirm the validity of this tool more objectively by measuring the change in resilience score after intervention by psychiatrist or psychology counselor. Second, test-retest reliability was not measured, which would help to confirm the test consistency. Both short-term and long-term test-retest reliabilities are required to verify test consistency. Third, the authors used only one psychometric indicator of PTSD. Results of average analysis by symptom group poorly demonstrated the statistical significance of IES-R. It has been reported that resilience is a protective factor against the development of PTSD and has an influential role in buffering the adverse effects of mental trauma [40, 41]. To confirm this, it is crucial to conduct a questionnaire that accurately reflects the symptoms of PTSD. Further studies with additional psychometric indicators of PTSD, such as the posttraumatic stress diagnostic scale or other well-made measures, are needed [42–44]. Fourth, this study did not include a control group. Since comparison between results of the control and this study might be important to confirm the validity of the scale, control group should be included in future studies.

## Conclusions

The study demonstrates that the 10-item K-CD-RISC provides useful psychometric properties and is a valid tool for assessing workers who experience environmental accidents such as toxic chemical exposure (HF).

## Abbreviations

BAI: Beck Anxiety Inventory
CES-D: The Center for Epidemiologic Studies of Depression Scale
IES-R: Impact of Event Scale-revised
K-CD-RISC 10: Korean Version of the 10-item Conner-Davidson Resilience Scale
PSS: Perceived Stress Scale
RSES: Rosenberg Self-Esteem Scale

## References

[1] Rutter M (1985). Resilience in the face of adversity: protective factors and resistance to psychiatric disorders. Br J Psychiatry.

[2] Norris FH, Stevens SP, Pfefferbaum B, Wyche KF, Pfefferbaum RL (2008). Community resilience as a metaphor, theory, set of capacities, and strategy for disaster readiness. Am J Community Psychol.

[3] Connor KM, Davidson JR (2003). Development of a new resilience scale: the Connor-Davidson resilience scale (CD-RISC). Depress Anxiety.

[4] Agaibi CE, Wilson JP (2005). Trauma, PTSD, and resilience: a review of the literature. Trauma Violence Abuse.

[5] Charney DS (2004). Psychobiological mechanisms of resilience and vulnerability: implications for successful adaptation to extreme stress. Am J Psychiatry.

[6] Hoge EA, Austin ED, Pollack MH (2007). Resilience: research evidence and conceptual considerations for posttraumatic stress disorder. Depress Anxiety.

[7] Bonanno GA (2004). Loss, trauma, and human resilience: have we underestimated the human capacity to thrive after extremely aversive events. Am Psychol.

[8] Bonanno GA, Galea S, Bucciarelli A, Vlahov D (2007). What predicts psychological resilience after disaster? The role of demographics, resources, and life stress. J Consult Clin Psychol.

[9] Southwick SM, Vythilingam M, Charney DS (2005). The psychobiology of depression and resilience to stress: implications for prevention and treatment. Annu Rev Clin Psychol.

[10] Ahern NR, Kiehl EM, Sole ML, Byers J (2006). A review of instruments measuring resilience. Issues Compr Pediatr Nurs.

[11] Windle G, Bennett KM, Noyes J (2011). A methodological review of resilience measurement scales. Health Qual Life Outcomes.

[12] Connor KM (2006). Assessment of resilience in the aftermath of trauma. J Clin Psychiatry.

[13] Campbell-Sills L, Stein MB (2007). Psychometric analysis and refinement of the Connor-davidson resilience scale (CD-RISC): validation of a 10-item measure of resilience. J Trauma Stress.

[14] Gucciardi DF, Jackson B, Coulter TJ, Mallett CJ (2011). The Connor-Davidson resilience scale (CD-RISC): dimensionality and age-related measurement invariance with Australian cricketers. Psychol Sport Exerc.

[15] Wang L, Shi Z, Zhang Y, Zhang Z (2010). Psychometric properties of the 10-item Connor-Davidson resilience scale in Chinese earthquake victims. Psychiatry Clin Neurosci.

[16] Burns RA, Anstey KJ (2010). The Connor–Davidson resilience scale (CD-RISC): testing the invariance of a uni-dimensional resilience measure that is independent of positive and negative affect. Personal Individ Differ.

[17] Jørgensen IE, Seedat S (2008). Factor structure of the Connor-Davidson resilience scale in south African adolescents. Int J Adolesc Med Health.

[18] Clauss-Ehlers CS (2008). Sociocultural factors, resilience, and coping: support for a culturally sensitive measure of resilience. J Appl Dev Psychol.

[19] Gillespie BM, Chaboyer W, Wallis M, Grimbeek P (2007). Resilience in the operating room: developing and testing of a resilience model. J Adv Nurs.

[20] Singh K, Yu XN (2010). Psychometric evaluation of the Connor-Davidson resilience scale (CD-RISC) in a sample of Indian students. J Psychol.

[21] Yu X, Zhang J (2007). Factor analysis and psychometric evaluation of the Connor-Davidson resilience scale (CD-RISC) with Chinese people. Soc Behav Personal Int J.

[22] Kukihara H, Yamawaki N, Uchiyama K, Arai S, Horikawa E (2014). Trauma, depression, and resilience of earthquake/tsunami/nuclear disaster survivors of Hirono, Fukushima. Japan Psychiatry Clin Neurosci.

[23] Ahmad S, Feder A, Lee EJ, Wang Y, Southwick SM, Schlackman E, Buchholz K, Alonso A, Charney DS (2010). Earthquake impact in a remote south AsianPopulation: psychosocial factors and posttraumatic symptoms. J Trauma Stress.

[24] Heetkamp T, Terte ID (2015). PTSD and resilience in adolescents after New Zealand earthquakes. N Z J Psychol.

[25] Grattan LM, Roberts S, Mahan WT, McLaughlin PK, Otwell WS, Morris JG (2011). The early psychological impacts of the Deepwater horizon oil spill on Florida and Alabama communities. Environ Health Perspect.

[26] Notario-Pacheco B, Solera-Martínez M, Serrano-Parra MD, Bartolomé-Gutiérrez R, García-Campayo J, Martínez-Vizcaíno V (2011). Reliability and validity of the Spanish version of the 10-item Connor-Davidson resilience scale (10-item CD-RISC) in young adults. Health Qual Life Outcomes.

[27] Baek HS, Lee KU, Joo EJ, Lee MY, Choi KS (2010). Reliability and validity of the korean version of the connor-Davidson resilience scale. Psychiatry Investig.

[28] Asukai N (2002). Reliability and validity of the Japanese-language version of the impact scale of event scale-revised (IES-R-J): four studies of different traumatic events. J Nerv Ment Dis.

[29] Lee KS, Kim DH, Cho YR (2018). Exploratory factor analysis of the Beck anxiety inventory and the Beck depression inventory-II in a psychiatric outpatient population. J Korean Med Sci.

[30] Beck AT, Epstein N, Brown G, Steer RA (1988). An inventory for measuring clinical anxiety: psychometric properties. J Consult Clin Psychol.

[31] Cho MJ, Kim KH (1998). Use of the Center for Epidemiologic Studies Depression (CES-D) scale in Korea. J Nerv Ment Dis.

[32] Radloff LS (1977). The CES-D scale as a self-report depression scale for research in the general population. Appl Psychol Meas.

[33] Westaway MS, Jordaan ER, Tsai J (2015). Investigating the psychometric properties of the Rosenberg self-esteem scale for south African residents of greater Pretoria. Eval Health Prof.

[34] Cohen S, Williamson GM, Spacapan S, Oskamp S (1988). Perceived stress in a probability sample of the United States. The social psychology of health.

[35] O’Connor BP (2000). SPSS, SAS, and MATLAB programs for determining the number of components using parallel analysisand Velicer’s MAP test. BehavRes Methods Instrum Comput.

[36] Weisæth L (1986). An industrial disaster. Disaster behavior and posttraumatic stress reactions. TidsskrNorLaegeforen.

[37] Weisæth L (1989). Importance of high response rates in traumatic stress research. Acta Psychiatr Scand.

[38] Weisæth L (1989). The stressors and the posttraumatic stress syndrome after an industrial disaster. ActaPsychiatricaScandinavica.

[39] Kolltveit S (2012). Risk factors for PTSD, anxiety, and depression among adolescents in Gaza. J Trauma Stress.

[40] Bibi A, Kalim S, Khalid MA. Post-traumatic stress disorder and resilience among adult burn patients in Pakistan: a cross-sectional study. Burns Trauma. 2018;6:8. 10.1186/s41038-018-0110-7 PMC6040608 30009193

[41] King LA, King DW, Fairbank JA, Keane TM, Adams GA (1998). Resilience recovery factors in post-traumatic stress disorder among female and male Vietnam veterans: hardiness, postwar social support, and additional stressful life events. J Pers Soc Psychol.

[42] King LA, King DW, Leskin G, Foy DW (1995). The Los Angeles symptom checklist: a self-report measure of posttraumatic stress disorder. Assessment.

[43] Elhai JD, Gray MJ, Kashdan TB, Franklin CL (2005). Which instruments are most commonly used to assess traumatic event exposure and posttraumatic effects?: a survey of traumatic stress professionals. J Trauma Stress.

[44] Orsillo SM, Antony MM, Orsillo SM (2001). Measures for acute stress disorder and posttraumaticstress disorder. Practitioner’s Guide to Empirically Based Measures ofAnxiety.

